# Correction to: the following articles

**DOI:** 10.1093/procel/pwaf065

**Published:** 2025-08-25

This is a corrected-article to the following papers:

Yibo Wang, Huiyu Xu, Lin Fu, Yang Yu, Jie Qiao, A promising clinical technique to rescue women’s ovarian aging: engineered stem cell-based therapy with the help of AI, *Protein & Cell*, Volume 16, Issue 4, April 2025, Pages 233–238, https://doi.org/10.1093/procel/pwae047

Stefania Militi, Reshma Nibhani, Martin Pook, Siim Pauklin, SMAD2/3-SMYD2 and developmental transcription factors cooperate with cell-cycle inhibitors to guide tissue formation, *Protein & Cell*, Volume 16, Issue 4, April 2025, Pages 259–282, https://doi.org/10.1093/procel/pwae031

Ming-Heng Li, Xiaoyu Jiang, Yaobin Jing, Kaowen Yan, Shi-Jia Bi, Si Wang, Shuai Ma, Guang-Hui Liu, Weiqi Zhang, Shuhui Sun, Jing Qu, CRISPR-based screening pinpoints H2AZ1 as a driver of senescence in human mesenchymal stem cells, *Protein & Cell*, Volume 16, Issue 4, April 2025, Pages 293–299, https://doi.org/10.1093/procel/pwae035

Wenjing Na, Wenfeng Zeng, Kai Song, Youwang Wang, Luoyang Wang, Ziran Zhao, Lingtao Jin, Ping Zhu, Wei Liang, PKM2, the “K^+^ sink” in the tumor interstitial fluid, *Protein & Cell*, Volume 16, Issue 4, April 2025, Pages 300–306, https://doi.org/10.1093/procel/pwae036

Wei Han, Siyu Bao, Jintao Liu, Yiran Wu, Liting Zeng, Tao Zhang, Ningmeng Chen, Kai Yao, Shunguo Fan, Aiping Huang, Yuanyuan Feng, Guiquan Zhang, Ruiyi Zhang, Hongjin Zhu, Tian Hua, Zhijie Liu, Lina Cao, Xingxu Huang, Suwen Zhao, The chordata olfactory receptor database, *Protein & Cell*, Volume 16, Issue 4, April 2025, Pages 283–292, https://doi.org/10.1093/procel/pwae050

Wenwen Wang, Pu Liu, Wendi Zhu, Tianwei Li, Ying Wang, Yujie Wang, Jun Li, Jie Ma, Ling Leng, Skin organoid transplantation promotes tissue repair with scarless in frostbite, *Protein & Cell*, Volume 16, Issue 4, April 2025, Pages 239–258, https://doi.org/10.1093/procel/pwae055

The originally cited erroneous page numbers for these articles have been emended.

DOI pwae047 is emended to read 233–238 instead of 233–239; DOI pwae031 is emended to read 259–282 instead of 260–285; DOI pwae035 is emended to read 293–299 instead of 296–305; DOI pwae036 is emended to read 300–306 instead of 303–308; DOI pwae050 is emended to read 283–292 instead of 286–295; DOI pwae055 is emended to read 239–258 instead of 240–259.

